# Idiopathic lymphatic mesenteric cyst of the proximal jejunum: A case report of an unusual clinical presentation

**DOI:** 10.1016/j.ijscr.2022.107402

**Published:** 2022-07-09

**Authors:** Michele Ghielmetti, Kerstin J. Neuschütz, Anna Hirschmann, Katharina Marston, Daniel C. Steinemann, Marco von Strauss und Torney

**Affiliations:** a Clarunis, Department of Visceral Surgery, University Center for Gastrointestinal and Liver Diseases, St. Clara Hospital and University Hospital Basel, Basel, Switzerland; b Imamed, Radiology Northwest, Basel, Switzerland; c Viollier AG, Basel, Switzerland

**Keywords:** CT, computer tomography, PMP, pseudomyxoma peritonei, Mesenteric cyst, Lymphatic mesenteric cyst, Chylous mesenteric cyst, General surgery, Laparoscopy

## Abstract

**Introduction:**

Mesenteric cysts are rare lesions of the abdominal cavity or retroperitoneum. The exact etiopathogenesis is still undefined. Clinical manifestation can vary from asymptomatic patients to symptoms of an acute abdomen, making diagnosis very challenging.

**Case presentation:**

We present a case of a 47-year-old male with new ongoing polyuria and nocturia as well as episodes of slight abdominal pain. An abdominal ultrasound showed ascites and the computer tomography (CT) scan raised suspicion of an internal hernia. We performed a diagnostic laparoscopy and open resection of a cystic lesion of the small bowel mesentery. The histological examination revealed a lymphatic mesenteric cyst.

**Discussion:**

Mesenteric cysts represent rare intra-abdominal tumors that physicians should consider as a differential diagnosis in patients with abdominal pain and an intra-abdominal mass.

**Conclusion:**

Surgery should be advised to prevent the development of complications and to confirm the histopathological diagnosis.

## Highlights

• Idiopathic mesenteric cyst with an unusual clinical presentation
• Mesenteric cysts are rare but should still be considered as differential diagnosis.
• Surgery is only an option to clarify diagnosis.

## 1 Introduction

The etiology of abdominal pain, acute or chronic, includes a vast amount of conditions, some of which are very frequent and well known, other are infrequent or even rare.

Mesenteric cysts are rare intra-abdominal tumors with a reported incidence between 1/100,000 to 1/250,000 hospital admissions in adults [1], [2], [3], [4], and 1/20,000 in children [5].

Because of its low incidence, several definitions and classifications of mesenteric cysts have been proposed. Nowadays most authors define the term mesenteric cyst as a heterogeneous group of cystic formation located anywhere in the mesentery, omentum or retroperitoneum [1], [3], [5], [6], [7]. The specific pathogenesis remains undefined and multiple hypotheses have been suggested. One theory includes the proliferation of ectopic lymphatic tissue or embryonic lymphatic channels that lack communication with the main lymphatic system. Another hypothesis suggests a failure of fusion of the mesenteric leaves resulting in lymphatic fluid accumulation. It is also supposed that some lymphatic cysts may result from external trauma [2], [8].

Benevieni, an Italian anatomist, reported a mesenteric cyst for the first time. He described this lesion in 1507 in an autopsy on an 8-year-old boy. Rakitansky published the first accurate description of a chylous cyst in 1842. Tillaux carried out the first successful surgical resection in 1880 [6]. Since then, mesenteric cysts of different origin were increasingly reported, nevertheless remaining a rare cause of abdominal pain.

We present a case of a 47-year-old male patient with a lymphatic mesenterial cyst of the proximal jejunum. This case report has been reported in line with the SCARE criteria [9].

## 2 Case presentation

**Fig. 1:**
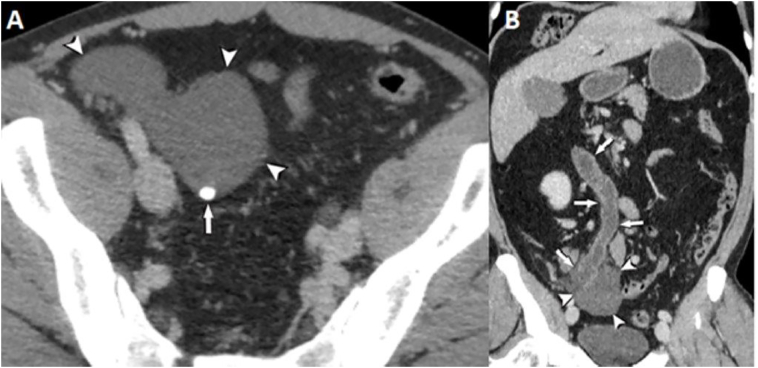
A + B: Axial (A) and coronal (B) enhanced CT images show localized fluid in the right lower abdominal quadrant (arrowheads in A and B) with a solitary calcification (arrow in A). The proximal slightly fluid-distended jejunal intestine loop (arrows in B) has an atypical straight orientation towards the right lower abdomen with swirling mesentery vessels suspicious for an internal hernia.

A 47-year-old male patient initially presented new urological symptoms such as polyuria and nocturia. Furthermore, he reported episodes of slight abdominal pain and a pre-existing gastroesophageal reflux. The urological examination revealed a benign prostatic enlargement without signs of urethral obstruction. An abdominal ultrasound showed ascites, other intra-abdominal pathologies were not described. A contrast-enhanced computed tomography (CT) scan of the abdomen was performed for further investigation (Fig. 1A + B). It confirmed the ascites, and additionally showed focal calcification between small bowel loops in the right lower abdominal quadrant. Furthermore, the CT scan raised suspicion of an internal hernia at the same place. However, there were no signs of a complete or incomplete small bowel obstruction.

The patient was referred to our institution for surgical consultation. The physical examination remained unremarkable with no sign of an abdominal mass, abdominal tenderness, or peritonitis. A recent colonoscopy a year earlier had shown no significant findings. The personal history to that point remained unremarkable as well with no previous abdominal surgery or medication. The lab results showed no abnormalities.

Due to the unclear findings, the potential internal hernia, the possible differential diagnosis of a pseudomyxoma as the origin of the unclear calcification and the ascites, surgery was planned. A diagnostic laparoscopy was performed by an attending and a surgical resident. It showed no sign of ascites or an internal hernia. Yet, we found a cystic lesion with perifocal milky fluid accumulation, located in the mesentery of the proximal jejunum. We performed a mini-laparotomy and a complete excision of the lesion. Due to the proximity of the lesion to the small bowel, we performed an “en bloc” intestinal loop resection of about six centimeters and an end-to-end anastomosis.

**Fig. 2:**
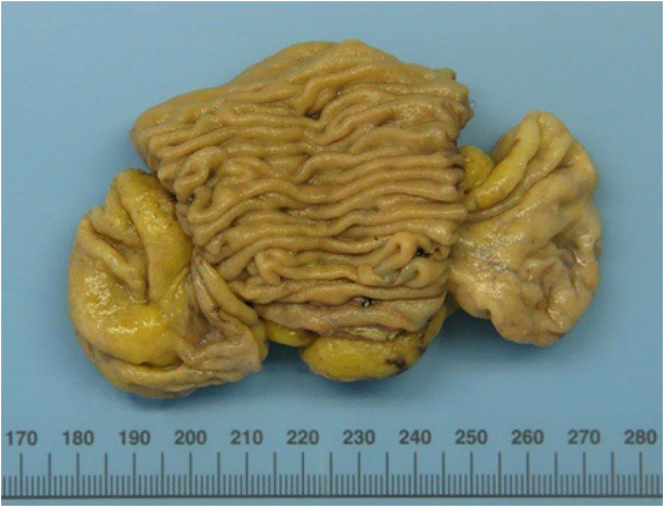
Macroscopic image of the lymphatic mesenteric cyst (pathological specimen).

**Fig. 3:**
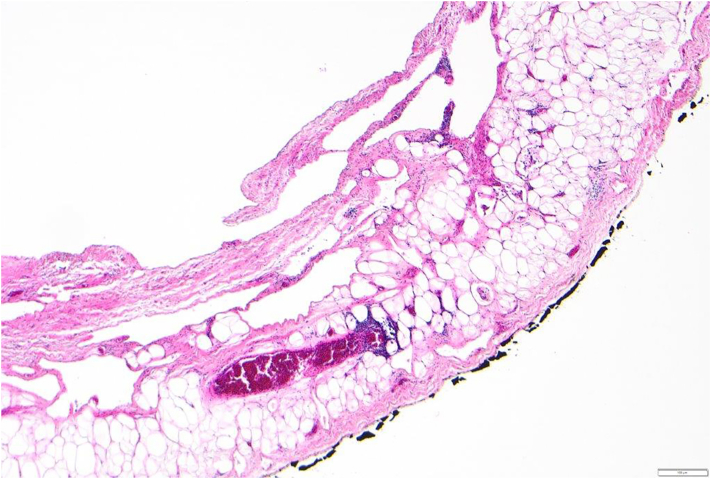
Microscopic image of the lymphatic mesenteric cyst.

The histopathological examination confirmed a lymphatic mesenteric cyst with a diameter of 11 cm (Fig. 2, Fig. 3). There was no contact between the cyst and the small bowel.

No postoperative complications occurred. The patient remained at fasting for the first three postoperative days and there was no complication during the following oral food intake. The patient was discharged on full diet and without any complication on the ninth postoperative day. On the 6-week follow-up the patient was doing well and mentioned no discomfort or abdominal pain.

Retrospectively, the patient remembered a hospitalization over several days due to unspecific abdominal pain, nausea and vomiting at the age of 10. The pain was located in the epigastric area and was also associated with abdominal cramps and incapacity of oral food intake for several days. The diagnostic assessment carried out during that hospitalization remained inconclusive but no abdominal imaging had been available at that time. The patient also reported, he suffered from several similar episodes of the same unspecific abdominal pain during his teenage years until they resolved at the beginning of adulthood.

## 3 Discussion

The clinical manifestation of mesenteric cysts is mostly non-specific and can present with various symptoms. As in our case, up to 40 % of mesenteric cysts [2] are oligo-symptomatic and are diagnosed during surgery or while diagnostic assessment for a different reason.

The symptoms can vary from non-specific, chronic abdominal pain to symptoms of an acute abdomen mimicking rupture of an abdominal aortic aneurysm [8].

In patients with non-specific, usually chronic diffuse abdominal pain, symptoms are thought to be caused from traction and stretching on the root of the mesentery [2]. Giant mesenteric cysts may produce extrinsic compression of the bowel resulting in intermittent nausea, vomiting, changes in bowel habits due to intestinal obstruction.

Acute symptoms are usually related to complications and may include hemorrhage, shock, rupture, volvulus or bowel infarction, resulting in surgical emergency situations [1], [2], [10].

Mesenteric cysts can also present clinical or radiological findings similar to malignant lesions such as appendiceal cancer, most of all pseudomyxoma peritonei (PMP). CT imaging of our patient, showing focal calcification and fluid collection between small bowel loops in the right lower abdominal quadrant, also aroused the suspicion of PMP and led to consider a diagnostic laparoscopy.

The etiopathogenesis of mesenteric cysts is still unknown. Hypotheses include traumatic, infectious, or embryonic origins [11]. Some authors hypothesize that episodes of intermittent volvulus may result in lymphatic obstruction and thereby lead to the formation of a lymphatic cyst [12]. A possible explanation for the development of the cyst in our patient might be a severe infection or enteritis in childhood leading to an interruption of the lymphatic flow in the proximal jejunum.

As reported by several authors, most mesenteric cysts are located in the small bowel mesentery (about 60 % of cases), followed by the large bowel mesentery (24 %), and the retroperitoneum [3], [4], [5]. In our case, the cyst was located in the mesentery of the proximal jejunum and thus, in the most common location mentioned in the literature.

As mentioned before, several definitions and classifications have been proposed. The most accepted classification nowadays divides mesenteric cysts according to their histopathological features. This classification includes the 6 following groups: cysts of lymphatic origin, mesothelial origin, enteric origin, urogenital origin, mature cystic teratomas and pseudocysts. [1], [6]. Lymphatic mesenteric cyst was the histopathological diagnosis of the case we presented above. These cysts are filled with either serous or chylous fluid. Cysts with serous content are mostly located in the mesocolon, whereas chylous-filled cysts are mostly found in the mesentery of the small bowel [2], as observed in our patient.

Surgery is the treatment of choice for this kind of lesion, although good results have been reported with percutaneous drainage and sclerosis of the cystic wall [13], [14]. Partial excision and cyst-deroofing have also been reported. This management, drainage in particular, is associated with high rate of recurrence and should therefore be avoided [8]. Complete surgical resection should be performed not only to prevent recurrence, but also to avoid related complications or malignant transformation [1], [6]. Malignant neoplasms ongoing from the cyst wall as well as primary malignant cysts are very rare, but cases of sarcoma and adenocarcinoma have been reported [2], [4], [5], [6].

If feasible, laparoscopy should be preferred to open surgery to reduce postoperative pain, need for analgesic drugs and postoperative length of hospital stay.

## 4 Conclusion

Mesenteric cysts are a rare entity. Nevertheless, they should be considered as a differential diagnosis as the clinical features may be unspecific. Complete surgical resection is the best option to confirm the diagnosis and prevent recurrences.

## Consent

Written informed consent was obtained from the patient for publication of this case report and accompanying images. A copy of the written consent is available for review by the Editor-in-Chief of this journal on request.

## Provenance and peer review

Not commissioned, externally peer-reviewed.

## Ethical approval

N/A.

## Funding

This research did not receive any specific grant from funding agencies in the public, commercial, or not-for-profit-sectors.

## Guarantor

MvSuT.

## Research registration number

N/A.

## CRediT authorship contribution statement

All authors contributed to the article. The first draft of the manuscript was written by MG and MvSuT. KJN and DCS critically reviewed previous versions of the manuscript. AH obtained and interpreted the imaging and provided Fig. 1. KM performed the histopathological analyses and provided Fig. 2, Fig. 3. All authors approved the submitted version, agree to be accountable for their contributions and ensure the accuracy and integrity of the work.

## Declaration of competing interest

The authors have no conflicts of interest to declare.
